# Analysis of the Evolution of User Emotion and Opinion Leaders’ Information Dissemination Behavior in the Knowledge Q&A Community during COVID-19

**DOI:** 10.3390/ijerph182212252

**Published:** 2021-11-22

**Authors:** Xu Xu, Zhigang Li, Rui Wang, Li Zhao

**Affiliations:** 1 School of Economic Information Engineering, Southwestern University of Finance and Economics, Chengdu 611130, China; xuxujy@yeah.net (X.X.); lizhao@stu.cdut.edu.cn (L.Z.); 2 School of Management Science, Chengdu University of Technology, Chengdu 610059, China; rwangjy@163.com

**Keywords:** social network analysis, essence posts, knowledge Q&A community, information dissemination network, opinion leader, emotional evolution

## Abstract

Since its emergence in 2019, COVID-19 has quickly triggered widespread public discussion on social media. From 26 February 2020 to 26 September 2020, this study collected data on COVID-19-related posts in the knowledge Q&A community, identified 220 opinion leaders of this community, and used social network analysis and sentiment analysis to analyze the information exchange behavior and emotional evolution of the opinion leaders during COVID-19. The results show that the COVID-19 topic community could be divided into seven main categories. The information dissemination of opinion leader information dissemination network had low efficiency, multiple paths, and a high degree of control. In addition, the emotional evolution of users showed obvious phased characteristics. User emotion changed from initially strong negative to strong positive over the course of the pandemic and eventually tended to be objective and neutral as time passed and the event stabilized.

## 1. Introduction

Since its emergence at the end of 2019, the novel coronavirus (COVID-19) has been shown to be highly infectious [1], to spread quickly [2], to have multiple transmission routes [3], and to have universal susceptibility [4]. The World Health Organization declared COVID-19 a Public Health Emergency of International Concern on 30 January 2020 [5]. The COVID-19 epidemic has posed a huge challenge and threat to global public health and has accordingly become a focus of worldwide attention [6]. At the same time, information about COVID-19 has also spread rapidly on social media.

In recent years, social media has flourished on the basis of Internet technology [7], becoming an indispensable information channel for many people [8]. Social media provides a space for emotional venting and the collision of ideas, making it an amplifier of public opinion [9]. Trends in public opinion are known to affect social stability [10]. The COVID-19 epidemic has aroused widespread public discussion on social media. Online news related to the epidemic continues to increase [11], rumors frequently spread, and the expression of public opinion remains intense [12]. In the process of information dissemination on social media, the public can be highly susceptible to the content that is presented [13], and negative public opinion can arise as a result. Thus, it is important to understand the focus of public attention and effectively control and guide the healthy development of public opinion. This can help the government and health departments to better communicate with the public on important health issues. In the context of COVID-19, the management of public opinion on social media has become an urgent problem.

Exploring public discussion themes and using social network analysis to investigate and visualize information dissemination on social media can provide new ideas for addressing such problems. Many studies have investigated information dissemination on social media. In terms of information dissemination networks, many studies have focused on the main body of public opinion [14]. Adopting information dissemination theory and motivation theory, some scholars found that emotional motivation, the social motivation of the information received, and the credibility of the information source had positive effects on users’ communication intentions; meanwhile, the emotional tendency of information had a negative effect [15].

Social network analysis has been widely used to analyze the structure of information dissemination networks [16,17], key figures in information dissemination [18,19], and online public opinion topics [20]. Regarding social media applications, studies of the dissemination of public opinion information have focused on platforms such as Weibo [21,22,23], WeChat [24,25], Blogger [26,27], Instagram [28,29], Twitter [30,31], and Facebook [32,33]. At present, there are few documents in the literature regarding the analysis of information dissemination behavior in the knowledge Q&A community, but these studies still show that the social network analysis method has certain applicability in the community. In addition, users tend to pay more attention to informational and emotional attributes when forwarding information [34]. This feature is particularly prominent in public crisis events [35].

This study used the knowledge Q&A community to study the information transmission network of a topic community. This is the first time China’s knowledge Q&A social media platform (i.e., Zhihu) has been used to study public attention and information dissemination related to COVID-19. Moreover, we innovatively combined indicators to analyze information dissemination, including dissemination efficiency, the dissemination path, and the degree of control over dissemination. Additionally, we extended the emotional cognition theory to this category, focusing on the analysis of the emotional evolution process of community users. This study aimed to describe the information dissemination of this community during COVID-19, explore the characteristics of the information exchange behavior of opinion leaders in this community, and explain the characteristics and laws of user emotion evolution. The results can provide a better understanding of the public’s demands and emotions, provide support for relevant departments to better communicate with the public, suggest measures for managing information dissemination, and reducing the spread of rumors.

The rest of this paper is structured as follows: First, we introduce the theoretical background of this study and present a literature review. Next, Section 3 describes the data collection procedure, followed by the data analyses and the results of the study. Finally, this study concludes with a discussion of its implications, as well as an acknowledgment of its limitations.

## 2. Theoretical Basis and Concept Definition

### 2.1. Network Opinion Leaders

Network opinion leaders are people who have leadership ability and play a core role on the Internet platform. They are activists who often provide information, opinions, comments, and influence to others in interpersonal communication networks [36], and they also play a crucial role as intermediaries in the information network constituted by interpersonal communication [37]. The network opinion leaders are educated, communicatively competent, politically knowledgeable, and participatory as compared to other groups [38]. They accumulate their social interaction ties through different routes, such as self-identity, knowledge contribution, and reciprocity [39]. Opinion leader mining is an important topic of social network research that is of great significance in terms of internet public opinion control and information dissemination [40]. To this end, many scholars have adopted relational data [41], k-clique clustering [42], PageRank measure [43], network analysis [44], and other methods to identify and analyze opinion leaders in the network. However, unlike other social media, users in the knowledge Q&A community mainly seek information or knowledge from content creators through search, inquiry, and interaction [45]. Therefore, when identifying online opinion leaders in the knowledge Q&A community, we considered more the mutual influence and interaction between users, that is, whether the user regularly provides information, opinions, and comments to other users in the topic community and whether the user has an impact on other users.

### 2.2. Knowledge Q&A Community

There is a wide variety of contemporary mainstream social media, but knowledge Q&A communities have their own unique characteristics. First, these communities are divided into two categories: knowledge communities and social communities. Knowledge communities exchange knowledge and information through questions and answers, while social communities are mainly developed through user interaction and offline salon gatherings [46], and the above two forms have been gradually accepted in recent years. Examples of such knowledge Q&A communities include Quora, Yahoo! Answers, and Stack Overflow in the US, Knowledge-iN in Korea, and Baidu Know, Himalaya FM, and Zhihu in China. Related reports [47] show that the public has embraced and frequently engaged in the exchange of information in Q&A communities. Second, unlike other media, knowledge Q&A communities create a user-centered information exchange environment [48] where users seek information or knowledge from content creators primarily through searching, asking, and interacting [49,50]. Users can also obtain more valuable information in a back-and-forth Q&A based on their interests with other users or content creators in the community under the posts they are interested in [51]. In addition, users can also post their concerns and questions in various communities to obtain answers [52].

### 2.3. Emotional Cognition Theory

Emotional cognitive theory is derived from social psychology and is a branch of emotional theory. The theory proposes that after the body’s information obtained from the outside enters the perceptual system, it will be organized and compiled by the perception and perceptual system on the one hand, and on the other hand, the information will trigger the body’s positive or negative emotional reactions, which in turn triggers certain behavioral tendencies [53]. In previous studies, the analysis of user information dissemination in the knowledge Q&A community has mostly been based on the degree of knowledge contribution [54] and social exchange theory [55], social capital theory [56], answer selection model [57], and other theories or models. Some scholars attribute the information behavior of users in the community to knowledge exploration and the result of rational thinking. However, people are emotional creatures, and they are individuals who are extremely easily affected by emotions. Their information dissemination behavior is determined by the information they see and their own emotions. In addition, the user’s cognition and emotion in the process of reading information will play a role in the processing of information [58]. Through the body’s perception, users in the community will show obvious emotional tendencies when posting, which is in line with the basic characteristics of the emotional cognitive theory. Therefore, this research combines the emotional cognition theory with the analysis of community information dissemination behavior, which not only expands the scope of application of the theory but also focuses on understanding the emotional change trend of users in the knowledge Q&A community during the pandemic through the analysis of the temporal and spatial emotional evolution of users.

## 3. Materials and Methods

### 3.1. Procedure

There were four main steps in this research: (1) Collect essential posts from the COVID-19-themed community on Zhihu. (2) Based on the title text of the posts, identify COVID-19-related topic hotspots in the community through word frequency and word-cloud analysis. (3) construct an information dissemination network based on the information interaction behavior of opinion leaders and measure the network in terms of dissemination efficiency, the dissemination path, and the degree of control over dissemination. (4) The information on the percentage of new posts added daily is used to understand the user emotion evolution in public health emergencies. Each step is described in detail below.

### 3.2. The Sample

The sample came from Zhihu (www.zhihu.com) (accessed on 8 October 2021), which is an online knowledge Q&A community that triggers users to discuss issues through behaviors such as posting. Other users may then express their opinions through approval or comments. The sample came from Zhihu (www.Zhihu.com) (accessed on 8 October 2021), which is an online knowledge Q&A community that triggers users to discuss issues through behaviors such as posting. Other users may then express their opinions through approval or comments. Zhihu is an original content platform where high-quality Q&A, knowledge-sharing communities, and various creators gather. The market scale of the Zhihu online community has always maintained a growth trend higher than that of other content markets, reaching CNY 275.8 billion in 2019 and is expected to increase to CNY 1.3 trillion by 2025, with a compound annual growth rate of 30.3%. From the official launch of Zhihu in January 2011 to 2021, Zhihu APP MAU reached 67 million, the number of devices used per month was 485 million, and the average daily search volume was 25.45 million. The main users of Zhihu are creators (mainly refers to industry elites, self-media, Zhi and users) and ordinary users (mainly refers to the knowledge-based middle class, quality life seekers, Generation X). From the perspective of gender, age, and geographical dimensions, Zhihu’s user composition shows a diversified distribution trend and the characteristics of young and high consumption [59]. In general, Zhihu is a very representative and well-developed platform in the knowledge Q&A community.

Therefore, this study focused on the COVID-19 topic community in Zhihu. Data collection spanned from 26 February 2020 to 26 September 2020. The number of people who followed topics reached 9665, and the number of Q&A posts within topics reached 13,313. These figures continue to increase. Due to the poor relevance of common posts [60], for representativeness, the essence of the topic was captured, including the title, content, user name, number of approvals, number of comments, and related topic comments. We initially gathered 2047 essential posts and finally obtained 1890 after removing repetitive, redundant, or meaningless posts. We also identified the opinion leaders who posted the essential posts and gathered data on their characteristics, such as the number of answers, questions, articles, ideas, approvals, likes, favorites, and followers. These two sets of data were used for further processing and analysis.

### 3.3. Data Processing

Longseo and ROST-CM6 were used to process the word segmentation of the titles (in Chinese) of the 1890 posts. ROSTCM6 was developed by the ROST virtual learning team of Wuhan University. It is a text analysis software that includes character frequency statistics, word segmentation, Chinese word frequency statistics, sentiment analysis, and many other analysis functions [61]. Longseo is a Chinese word segmentation processing software whose main function is to extract the core words of the text. Therefore, we first extracted the core words of the text using Longseo from the text and merged similar words such as “new crown”, “pneumonia” and “COVID-19”. Based on the preliminary results of longseo processing, the user-defined word segmentation thesaurus in ROSTCM6 was modified, synonyms were merged, some high-frequency words (e.g., auxiliary words, function words, conjunctions, prepositions) that were not closely related were filtered, and some proper nouns (e.g., region, country, medicine) were added. Then word segmentation was performed on the text to ensure accuracy. At the same time, Word Art and Tableau 10.5 were used for word-frequency analysis and word-cloud production. An inductive thematic analysis was conducted on the discussion threads [62]. We read the discussions read multiple times, and then relevant posts were independently coded line by line by two authors, and the coding was iteratively discussed and refined after completion to ensure a rich and diverse interpretation of the data. Finally, we crawled the daily new posts, combined the sentiment classification model, and used ROSTCM6 to profile the user emotion evolution during major epidemics.

### 3.4. Data Analysis

#### 3.4.1. Variables and Instruments Used to Construct Networks

Social network construction was based on whether opinion leaders in the COVID-19 topic community had exchanged and shared information. We obtained the information exchange between opinion leaders and coded them using numbers to ensure privacy. The following social network analysis measures were used to analyze the social network of opinion leaders: density, average distance, centrality, core–periphery, and structural holes.

Density: the proportion of possible relationships in the network. The value ranges from 0 to 1 [63]. When n is the number of individuals in the network, and l is the number of lines that exist between users, it is expressed as:

(1) $$Density=\frac{l}{n(n-1)}$$

Average distance: the shortest path between two individuals, representing the degree of connection in the network [64].

Centrality: includes Freeman’s degree, Freeman’s betweenness, and closeness, which measure the interactions between individuals [65]. In the information dissemination network, $A=X_{ij}$, $g_{jk}$ represents the number of the shortest paths from node j to k, $g_{jk}(i)$ is the number of nodes i in the shortest path from j to k, and $d_{ij}$ represents the distance of the geodesic. The expressions for Freeman’s degree, closeness, and Freeman’s betweenness are as follows:

(2) $$Freeman’s\;degree\;centrality=\sum_{j}^{}X_{ij}$$

(3) $$Freeman’s\;betweenness\;centrality=\sum_{j<k}\frac{g_{jk}(i)}{g_{jk}}$$

(4) $$Closeness\;centrality=\frac{1}{\sum_{j}^{}d_{ij}}$$

The core–periphery model examines the location characteristics of individuals in the network [66].

The structural hole measures whether an individual is in a position of direct connection and whether the individual plays a key connecting role [67]. At the same time, individuals located in structural holes have high information control ability [68] and can obtain redundant information in the network [69]. It includes four measurement indicators: effective, efficiency, constraint, and hierarchy.

#### 3.4.2. Identification and Measurement of Opinion Leaders

We identified network opinion leaders through the mutual influence and interaction between users. The H-index, proposed in 2005, was chosen as the most suitable surrogate impact metric [70], which can also be used to describe and assess the magnitude of users’ influence in the network and the amount of output [71]. The degree of attention, interaction, and conversation content obtained through user’s posting of posts were used as the basis for measuring the influence of the poster [72], which was specifically reflected in the number of likes and comments on the post. The above two indicators can indirectly indicate the degree to which other users pay attention to or like the essence posts [73]. Based on the core idea of the H-index, the influence index of the posting user-*n* is $\left(x_{n1},x_{n2}\right)$, where $x_{n1}$ is the number of likes posted by user-*n*, and $x_{n2}$ is the number of comments posted by user-*n*. The expression is as follows:

(5) $$H-Index(n)=(x_{n1},x_{n2})\begin{array}{cc} & n=1,2,3,\ldots ,m \end{array}$$

#### 3.4.3. Measurement of Network Information Dissemination Ability

We aimed to measure the information dissemination ability of the entire network according to the efficiency, path, and control degree of information dissemination. Information dissemination efficiency is mainly measured by density, distance, and network cohesion, which can effectively evaluate the flow of information in the entire network [74]. Analyzing the propagation path of an information dissemination network is mainly done by evaluating the depth of individual influence in the network [75], which is mainly measured by centrality. Individuals in the critical path can play a role in maintaining the order of information dissemination in the community. The user’s degree of control over information refers to the likelihood of the information being transmitted to other users [76]. In the process of information dissemination, the more information that passes through a user, the greater the user’s control over information dissemination, the greater the influence of the node in the information dissemination process, and the greater the trust. In this study, this was mainly measured through structural holes and the core–periphery model.

## 4. Results

### 4.1. Thematic Analysis

#### 4.1.1. Word Cloud and Word Frequency

**Figure 1:**
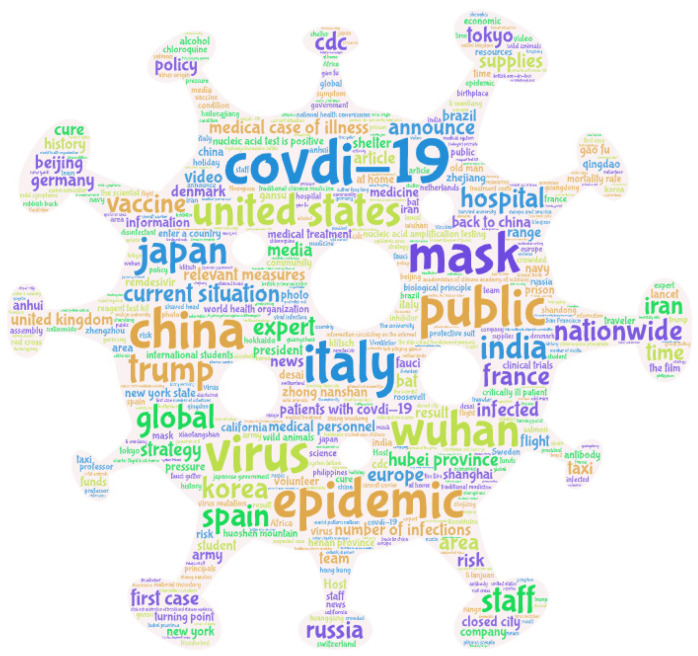
Word-cloud diagram of essential posts in the COVID-19 topic community.

**Figure 2:**
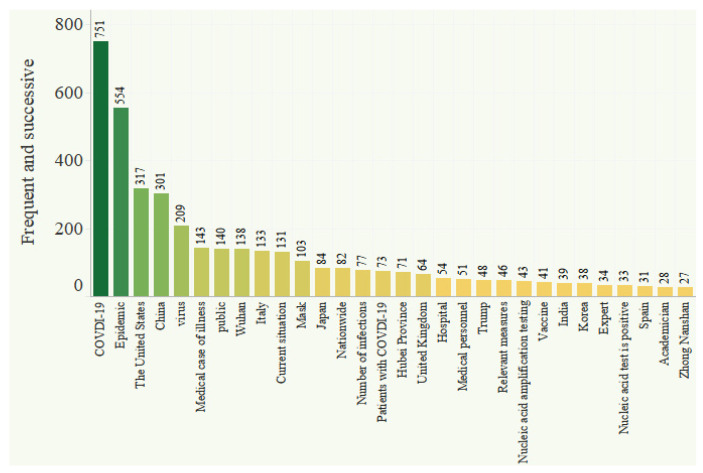
Word frequency in essential posts in the COVID-19 topic community (top 20).

Figure 1 shows a word-cloud diagram of the titles of the essential posts. The size of a word indicates its frequency. Figure 1 and Figure 2 show that “COVID-19” appeared in the titles of essential posts 751 times, followed by “epidemic” (554 times), “United States” (317 times), and “China” (301 times). These were the hottest words in the topic community. In terms of areas of concern, Hubei, Henan, and Beijing, where the epidemic had been the most severe in China, were a focus of discussion. Regarding regions outside of China, discussion hotspots included the US, Italy, Japan, South Korea, the UK, and Germany. Other areas of focus included masks, medicine, other COVID-19-related materials, and confirmed cases domestically and internationally.

#### 4.1.2. Classification of Essential Posts

**Figure 3:**
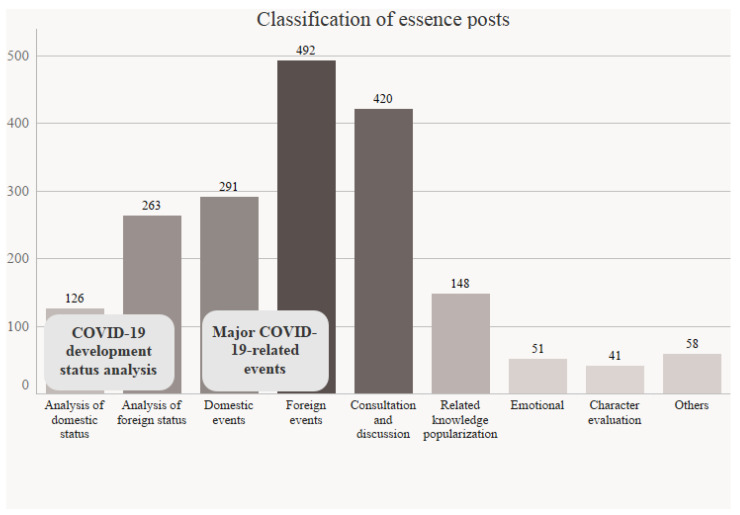
Classification statistics of essential posts in the COVID-19 theme community.

To classify the topics of essential posts, the 1890 essential posts were divided into seven categories according to content. Then, we counted the number of essential posts of each type (Figure 3). We found that 783 posts concerned COVID-19-related events, tending to focus more on foreign rather than domestic events. There was a total of 389 elite posts involving COVID-19 development status analysis, again with more focus on non-Chinese contexts. Meanwhile, 148 posts (just 7.83%) focused on promoting science and refuting rumors about drugs and treatment. Consultation and discussion posts accounted for 22.22%, suggesting that the public had some doubts and concerns regarding COVID-19.

### 4.2. Identification of Opinion Leaders

After drawing a scatter plot according to the $H-index=(x_{n1},x_{n2})$ of each user, it was found that when $x_{n1}\geq 5000$ and $x_{n2}\geq 700$, there is an obvious layering phenomenon for posting users. Thus, we set the filtering conditions to 5000 likes and 700 comments or more and obtained a total of 245 posts after filtering. Excluding duplicate posts, a total of 220 Internet opinion leaders were obtained. Their user information was captured using crawler software. After removing duplicate and invalid information, nine fields were obtained to describe user characteristics (Table 1): number of answers, number of questions, number of articles, number of ideas, number of approvals, number of likes, number of favorites, number followed, and number of followers.

### 4.3. Information Dissemination Network Analysis

#### 4.3.1. Dissemination Efficiency

**Figure 4:**
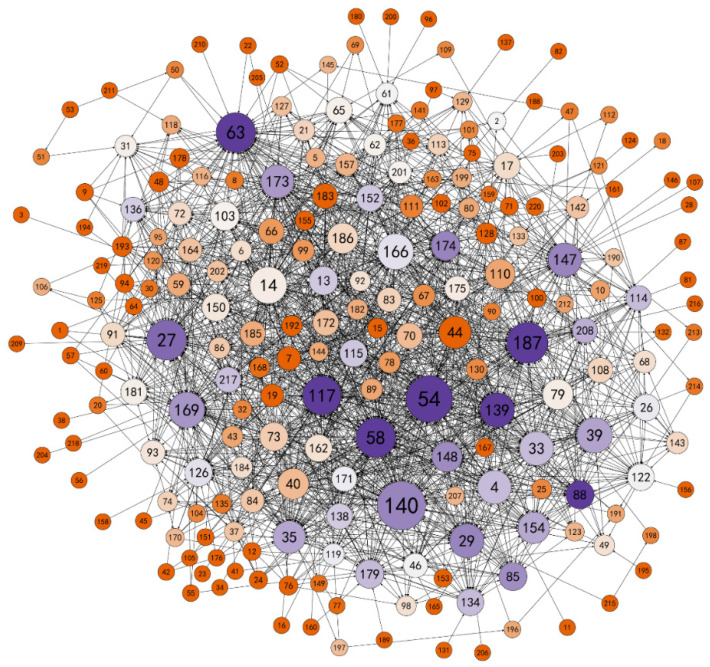
Diagram of opinion leaders’ information interaction network.

In Figure 4, which was created using Gephi, the nodes mainly rely on the degree of centrality in the data. The round nodes indicate the 220 opinion leaders, while the larger circular shapes represent individuals with a high degree of centrality. Lines represent the relational ties in information seeking, and arrows indicate the direction of information seeking. Figure 4 shows that there are no isolated nodes in the network, indicating that there was information-exchange behavior among the opinion leaders of the community. However, the overall density (0.041) and cohesion (0.246) of the network are relatively low, indicating that overall connections were loose. At the same time, the information dissemination efficiency of the network was relatively low, with an average distance of 2.922, indicating that each individual could communicate with other individuals after an average of three people in the network.

#### 4.3.2. Dissemination Path

The propagation path concerns the key nodes of information dissemination in the network according to depth of influence, which is mainly measured by the centrality of the network. Network centrality mainly includes three categories: Freeman’s degree, Freeman’s betweenness, and closeness. Freeman’s degree represents the number of connections with other nodes, which is divided into out-degree and in-degree. As shown in Table 2, in the opinion leader information exchange network, certain individuals (4, 14, 40, 44, 54, 79, 110, 140, 166, 186) have a larger in-degree and are therefore good at receiving information from other users. Some individuals (27, 35, 54, 58, 63, 117, 139, 140, 147, 169, 173, 174, 187) have a larger out-degree, which means they are willing to actively discuss topics with other individuals and are good at expressing their opinions. Meanwhile, Freeman’s betweenness measures the degree of control of resources by actors. Those individuals (14, 27, 29, 35, 39, 54, 58, 63, 110, 117, 140, 147, 152, 166, 169, 173, 187) with larger Freeman’s betweenness are the middlemen of the network; they have high social proficiency within the topic community and influence the dissemination of information and knowledge. Many individuals in the network need these “social influencers” to connect with other individuals. Furthermore, those individuals (7, 19, 44, 53, 183, 215, 220) with larger out-closeness had more contact with other individuals in the network; they are good at expressing their opinions and are less affected by information from middlemen. Thus, the information dissemination path of the network was not singular, and more opinion leaders were located on the critical path of the information dissemination network.

#### 4.3.3. Degree of Control over Dissemination

**Figure 5:**
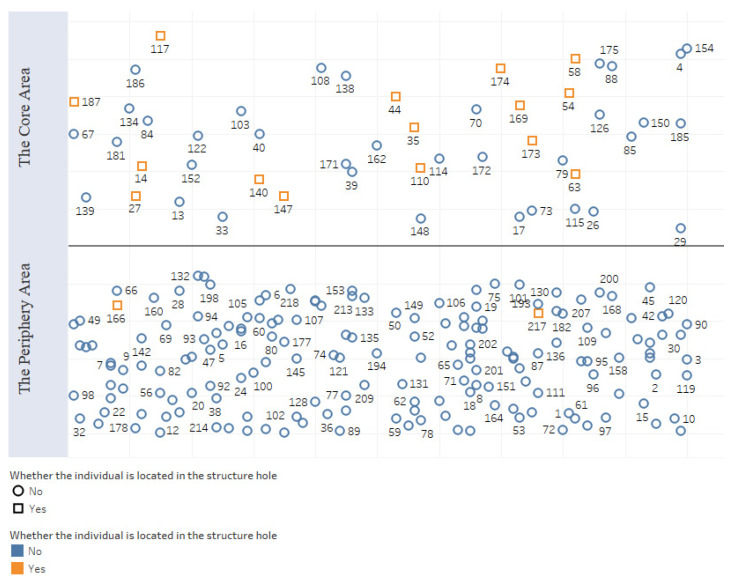
Distribution of opinion leaders’ core–periphery positions.

The degree of control over dissemination is measured by the number of structural holes in the network. Freeman’s betweenness indicated that there were structural holes in the network. By analyzing structural hole-related indicators, we found that certain individuals (14, 27, 35, 44, 54, 58, 63, 110, 117, 140, 147, 166, 169, 173, 174, 187, 217) were located in the structural hole of the network and were the middlemen of the network. Individuals at such nodes can also connect two areas to obtain nonredundant information in the network. There was also a core–periphery model in the network, and 52 individuals were in the core area. In Figure 5, the red-boxed individuals are in the structural hole of the network. More individuals with structural holes were in the core area of the network. They had greater access to information and thus had a competitive advantage over members in other positions. In general, due to the influence of the structural hole structure, the information dissemination of the network was hindered, and the degree of control over dissemination was relatively high.

### 4.4. The Evolution of User Emotion

**Figure 6:**
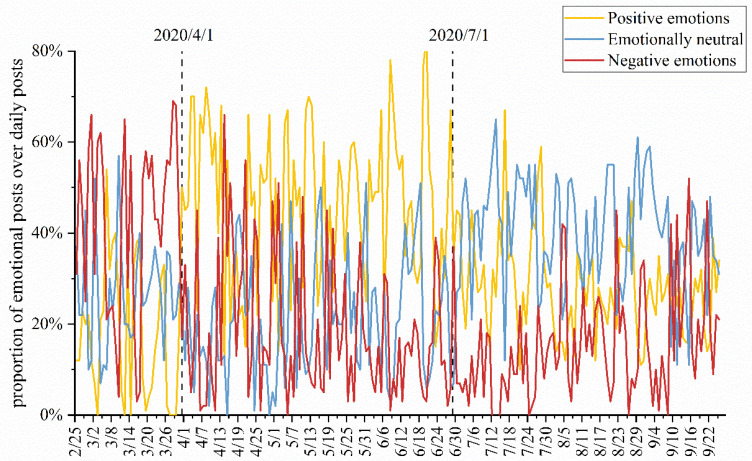
Proportion of emotional posts over daily posts.

From 26 February 2020 to 26 September 2020, we continuously crawled the daily new posts of this community over seven months and used ROSTCM6 to perform emotion analysis on the content of the posts. The study found that users’ emotional attitudes towards posting were divided into three main categories: positive, neutral, and negative. The process of user emotional evolution is mainly represented by the ratio of three daily emotion posts to daily posts, as shown in Figure 6. During the epidemic, the emotional evolution of users throughout the data collection period could be divided into three main phases. Before early April 2020, the three sentiments were relatively unstable, with the largest proportion of negative sentiment overall, which may be due to public concern and fear caused by uncertainty and ignorance about the initial outbreak of the epidemic. During the period from early April to the end of June 2020, the largest share of positive emotions was observed, while the share of neutral and negative emotions was fluctuating. Based on the actual situation, it was found that the domestic epidemic was gradually controlled and stabilized during this period, while the foreign epidemic started to break out continuously, and the national confidence in fighting the epidemic increased, and the positive emotions gradually increased. After 1 July 2020, the proportion of neutral emotions was generally highest among new daily emotional posts on COVID-19, with the user emotions stabilizing, and they responded to COVID-19 with a more objective attitude while discussion fever decreased. Overall, during the outbreak period of the epidemic, positive and negative emotions were dominant at separate times, while neutral emotions appeared during the dissipation of the epidemic.

## 5. Discussion and Conclusion

### 5.1. Discussion

Social media has played an increasingly prominent role in public health emergencies [77]. Discussions of COVID-19-related topics among users on knowledge Q&A social media show concentrated trends in terms of regions and topics. The essential posts collected for this study were classified into seven major categories, and there were significant differences in the number of posts in the different categories. In terms of major events and development status, users were more concerned about the international contexts than domestic ones; this may be caused by the uncertainty of COVID-19 in places such as Europe and the US [78]. We also found that discussion topics were closely related to the latest news and major events. Topics of interest have changed along with the development of the epidemic. It is noteworthy that a relatively small proportion of posts (7.83%) concerned the promotion of epidemic knowledge. Such knowledge can increase users’ awareness of COVID-19 and help reduce the spread of rumors [79].

We found that in the information interaction network, the information exchange between users was relatively loose (network density: 0.0414), and dissemination efficiency was relatively low. However, it had an average distance (2.992) and higher information accessibility. This differs from previous research conclusions [67], which could be attributable to differences between different social media in terms of user activity and event attention. Meanwhile, we identified 18 social influencers (higher Freeman betweenness) in the information dissemination network. These were the middlemen of information dissemination in the community. There were 23 information communication experts (higher out-degree or in-degree), who were good at expressing their views and communicating with others. This led to more information dissemination paths in the network, which was not singular. This also departs from previous findings [80], which could be attributed to the fact that academic Q&A communities have fewer users, less user-generated content, and a wider range of content dissemination than the research community. However, too many structural holes will lead to unconnected information transmission channels between users, resulting in high communication costs [81]. There were many structural holes in the topic community, leading to more control factors in users’ information exchange, which affects the transmission of information, making the network’s control over the degree of dissemination larger.

We also found an obvious core–periphery structure in the information exchange network of the community. In addition, the study also found that the density in the core area (0.297) was much higher than in the peripheral area (0.010). At the same time, individuals in the core area have higher information dissemination capabilities, and their information-exchange behavior will have a stronger effect on other individuals in the community. This is consistent with previous research [82,83], which shows individuals in the core area can obtain more diversified information, and their influence degree is greater than that of individuals in the peripheral area.

The results showed that user emotion changed from initially strong negative to strong positive over the course of the pandemic and eventually tended to be objective and neutral as time passed and the event stabilized. This means that in the early stages of an epidemic, information on social media about the worsening or rapid spread of the pandemic may cause public panic [84], as evidenced by a tendency for public opinion to be pessimistic. In addition, the severity of the epidemic is negatively correlated with the proportion of positive emotions and positively correlated with negative emotions. That is, as the epidemic worsens, people’s emotions shift to negative, and the proportion of positive emotions tends to decrease. This is not surprising at all, and the results of previous empirical studies confirm our results, showing stronger emotional fluctuations and higher psychological stress among the public during epidemics [85,86]. Interestingly, the introduction of government measures and the effective control of the epidemic have led to more positive user emotions, which manifests itself in social media in the form of more positive public opinion. Several scholars’ studies also prove this and argue that the government should deal with the epidemic in an open and objective manner to gain public trust, which also helps to reduce negative user emotions [87,88,89]. It is worth noting that as the epidemic subsides and the probability of major events decreases, public concern about the pandemic will gradually shift and mood swings will decrease, as evidenced by a decrease in the number of public comments on social media and a more neutral and objective evaluation of public opinion. In general, the evolution of public sentiment shows more obvious phased characteristics, which are closely related to the changes in the life cycle of public opinion.

### 5.2. Implications for Research and Practice

This research provides a new ideal to analyze the information dissemination behavior of users in the knowledge Q&A community. First, we extended the theory of emotional cognition to this field. After obtaining information in the community, through the body’s perception, it will trigger the user’s positive or negative emotional response. The research results also demonstrated that users have obvious emotional tendencies in the process of information dissemination. At the same time, the evolution of emotions had the characteristics of temporal and spatial changes. Secondly, we redefined the community’s online opinion leaders based on the unique characteristics of the knowledge Q&A community and the views of scholars. Combining communication studies and social network analysis methods to construct indicators, a quantitative analysis of the information dissemination network was carried out. The research results also confirmed that the user information dissemination network structure during the pandemic is different from the general state. All in all, user information dissemination behavior is a very complicated process, and researchers may need to focus more on the information dissemination mechanism and characteristics of users in the process of information dissemination.

In practice, first of all, in the face of a sudden pandemic, the results of this study have practical significance for effectively reducing the spread of malignant public opinion. The information dissemination of the opinion leader information dissemination network had low efficiency, multiple paths, and a high degree of control. Since the distortion and uncertainty of information will cause public panic, one effective way is to record their queries and invite expert members to respond to these questions. More importantly, more medium and small media accounts should be cultivated to increase the intensity of information diffusion, avoid information barriers caused by excessive concentration of information, and improve the efficiency of information dissemination.

Second, the results of this research also have practical significance for the emotional guidance of community users during the pandemic. We found that user emotions showed obvious phased characteristics. Thus, community managers could use information technology to actively pay attention to the development of characteristic groups in core areas and structural holes and identify some influential key people to release some positive information to significantly adjust and improve users’ negative emotions.

Third, this study also further reveals how managers could improve community functions to increase community utilization. On the one hand, some users with a high degree of knowledge contribution may be introduced to promote the flow of knowledge within the community. On the other hand, it is also necessary to increase the types of topic posts in the community, such as “Knowledge Popularization”, “Deed Sharing” and other related topics columns to help the members establish strong and long-term relationships with others and increase user stickiness.

### 5.3. Conclusions, Limitations, and Future Research

In this research, we obtained essential posts about COVID-19 on Zhihu. Based on the theory of emotional cognition, combined with the indicators of social network analysis, we constructed a social network for the information exchange behavior of opinion leaders and measured dissemination efficiency, dissemination path, and degree of control over dissemination. We also analyzed and explained the characteristics and laws of the evolution of user emotions in the knowledge Q&A community. The conclusions are summarized below. (1) Essential posts in the COVID-19 topic community could be divided into seven categories (e.g., major COVID-19-related events, consultation and discussion, development-status analysis of COVID-19, and knowledge popularization). (2) Opinion leaders in COVID-19-themed communities showed differences in their ability to disseminate information. The communication ability of individuals in the core area is significantly better than that of individuals in the peripheral area. (3) The information dissemination of opinion leader information dissemination network had low efficiency, multiple paths, and high degree of control. (4) During COVID-19, the emotional evolution of users in the knowledge Q&A community showed obvious phased characteristics. User emotion changed from initially strong negative to strong positive over the course of the pandemic and eventually tended to be objective and neutral as time passed and the event stabilized.

This study has some limitations. First, it was limited to the outbreak and stabilization period of COVID-19. Therefore, the situation before 26 February 2020 is beyond the scope of this work. Second, the data sources were relatively narrow. We only considered Zhihu and did not include other data sources such as Baidu Zhizhi and Douban. Therefore, in future research, we can collect users in different types of communities as samples to better analyze and reflect the network structure and behavior patterns of different users. This research was also limited to data disclosed by the platform. Furthermore, due to a lack of more detailed information about the users who contributed to essential posts, we were unable to describe users’ sociodemographic information, and we could not obtain the geographic spatial distribution of opinion leaders.

## Figures and Tables

**Table 1:** Information about opinion leaders (the top 20).

Node	Answers	Questions	Articles	Ideas	Approvals	Likes	Favorites	Followed	Followers
187	775	7	311	1064	4,427,729	876,161	3,371,310	308	2,961,288
117	3653	0	1212	642	7,632,933	944,971	2,205,389	127	2,776,704
17	214	8	22	32	498,041	317,217	925,781	905	89,391
129	14,243	0	830	982	3,491,598	365,752	921,719	38	264,810
173	1854	2	428	61	6,303,481	492,862	854,182	254	1,641,516
169	1508	6	40	949	5,476,721	535,175	840,646	79	799,315
201	249	0	455	0	2,183,119	240,481	806,312	19	298,173
212	2050	1	282	137	2,214,365	342,510	792,615	13	1,401,030
179	1897	297	1661	4758	999,646	164,500	665,398	1385	1,311,450
35	10,388	1390	160	2089	2,843,875	374,544	601,155	242	2,167,282
126	11,203	17	27	2603	3,375,730	35,792	584,300	257	304,892
186	163	0	86	12	759,608	132,906	533,675	552	334,760
54	1572	153	147	964	2,895,881	325,269	481,453	1141	1,016,351
13	751	8	47	1253	1,101,697	159,888	480,228	582	412,234
182	911	5	6	40	1,223,316	206,001	415,743	139	194,479
92	2400	1	25	13	1,155,062	163,414	399,878	44	198,812
174	829	4	18	33	929,442	157,933	374,313	118	403,985
63	447	17	333	514	2,536,188	172,221	374,150	193	949,990
68	741	19	40	721	916,109	122,278	365,038	273	513,848
58	252	0	31	137	770,716	123,994	345,119	95	875,600

**Table 2:** Centrality of the opinion leader information dissemination network.

Node	Freeman’s Degree		Freeman’s Betweenness	Closeness	
	Out-Degree	In-Degree		Out-Closeness	In-Closeness
4	32	25	931.471	1.141	42.442
7	25	0	0	1.153	0.455
14	40	27	1329.821	1.142	38.830
19	22	0	0	1.153	0.455
27	16	61	2250.844	1.140	48.667
29	19	39	1008.740	1.139	46.300
35	6	47	659.184	1.135	44.785
39	28	34	1100.431	1.141	44.603
40	39	9	735.769	1.142	37.694
44	52	0	0	1.183	0.455
53	2	0	0	1.166	0.455
54	38	59	3147.564	1.142	50.345
58	2	73	941.889	1.131	53.545
63	15	60	2071.452	1.138	48.026
79	32	15	552.177	1.141	37.889
110	37	7	573.174	1.142	34.488
117	4	68	1182.808	1.135	51.529
139	12	47	785.077	1.137	48.451
140	59	45	4782.494	1.143	47.198
147	15	41	1439.679	1.138	46.300
148	18	28	889.609	1.139	45.436
152	14	21	1055.198	1.139	38.693
166	37	27	2035.962	1.142	41.243
169	7	56	1146.631	1.138	46.695
173	10	45	758.197	1.137	45.342
174	1	41	408.984	1.123	47.505
183	29	0	0	1.154	0.455
186	30	17	440.518	1.141	38.153
187	21	59	1391.886	1.139	49.213
215	2	0	0	1.162	0.455
220	6	0	0	1.174	0.455
